# Phenotypic differences between *Drosophila* Alzheimer’s disease models expressing human *Aβ42* in the developing eye and brain

**DOI:** 10.1080/19768354.2017.1313777

**Published:** 2017-04-15

**Authors:** Youngjae Jeon, Soojin Lee, Myoungchul Shin, Jang Ho Lee, Yoon Seok Suh, Soojin Hwang, Hye Sup Yun, Kyoung Sang Cho

**Affiliations:** Department of Biological Sciences, Konkuk University, Seoul, Republic of Korea

**Keywords:** Alzheimer’s disease, amyloid-β42, *Drosophila*, *UAS-Aβ42*

## Abstract

*Drosophila melanogaster* expressing amyloid-β42 (*Aβ42*) transgenes have been used as models to study Alzheimer’s disease. Various *Aβ42* transgenes with different structures induce different phenotypes, which make it difficult to compare data among studies which use different transgenic lines. In this study, we compared the phenotypes of four frequently used *Aβ42* transgenic lines, *UAS-Aβ42^2X^*, *UAS-Aβ42^BL33770^*, *UAS-Aβ42^11C39^*, and *UAS-Aβ42^H29.3^*. Among the four transgenic lines, only *UAS-Aβ42^2X^* has two copies of the upstream activation sequence-amyloid-β42 (*UAS-Aβ42*) transgene, while remaining three have one copy. *UAS-Aβ42^BL33770^* has the 3′ untranslated region of *Drosophila α-tubulin*, while the others have that of SV40. *UAS-Aβ42^11C39^* and *UAS-Aβ42^H29.3^* have the rat pre-proenkephalin signal peptide, while *UAS-Aβ42^2X^* and *UAS-Aβ42^BL33770^* have that of the fly argos protein. When the transgenes were expressed ectopically in the developing eyes of the flies, *UAS-Aβ42^2X^* transgene resulted in a strongly reduced and rough eye phenotype, while *UAS-Aβ42^BL33770^* only showed a strong rough eye phenotype; *UAS-Aβ42^H29.3^* and *UAS-Aβ42^11C39^* had mild rough eyes. The levels of cell death and reactive oxygen species (ROS) in the eye imaginal discs were consistently the highest in *UAS-Aβ42^2X^*, followed by *UAS-Aβ42^BL33770^*, *UAS-Aβ42^11C39^*, and *UAS-Aβ42^H29.3^*. Surprisingly, the reduction in survival during the development of these lines did not correlate with cell death or ROS levels. The flies which expressed *UAS-Aβ42^11C39^* or *UAS-Aβ42^H29.3^* experienced greatly reduced survival rates, although low levels of ROS or cell death were detected. Collectively, our results demonstrated that different *Drosophila* AD models show different phenotypic severity, and suggested that different transgenes may have different modes of cytotoxicity.

**Abbreviations:** Aβ42: amyloid-β42; AD: Alzheimer’s disease; UAS: upstream activation sequence

## Introduction

Alzheimer’s disease (AD) is the most common neurodegenerative disorder and is characterized by amyloid plaques, neurofibrillary tangles, and loss of neurons (Mattson 2004). There are several hypotheses to explain the cause of AD (Hardy & Higgins 1992; Markesbery 1997; Francis et al. 1999; Hardy & Selkoe 2002; Berridge 2010; Maccioni et al. 2010). Among them, the amyloid hypothesis states that most of AD pathologies are caused by deposition of amyloid-β42 (Aβ42) peptide, which is generated by proteolytic processing of the amyloid precursor protein (Hardy & Higgins 1992).

**Figure 1.**
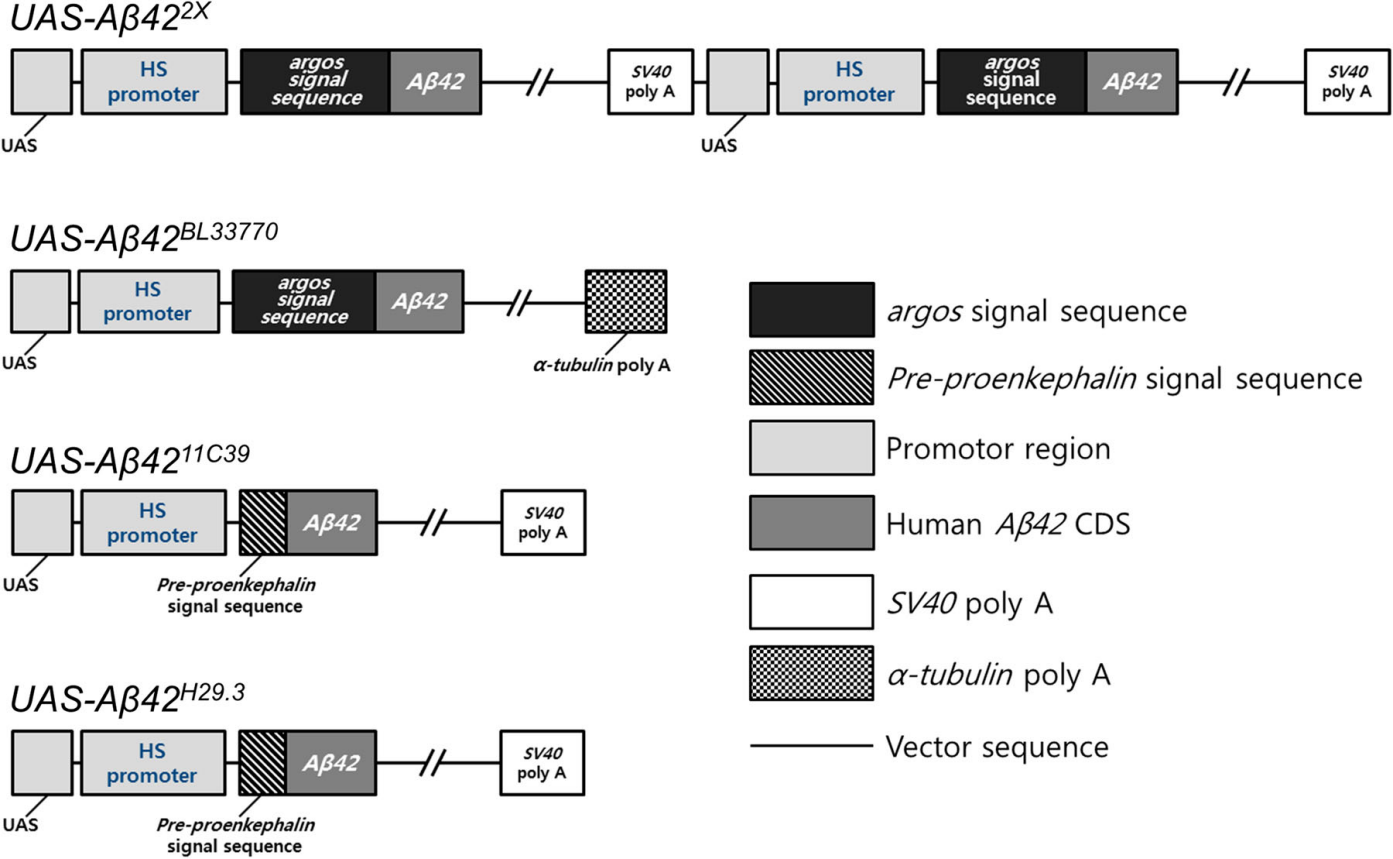
Constructs in four different *UAS-Aβ42* lines. The schematic figures show the constructs in the four *UAS-Aβ42* lines, *UAS-Aβ42^2X^*, *UAS-Aβ42^BL33770^*, *UAS-Aβ42^11C39^*, and *UAS-Aβ42^H29.3^*, which have differences in the number of copies, signal peptides, and poly A tails. *UAS-Aβ42^2X^* has two copies of the *UAS-Aβ42* sequence, while the others have one copy. *UAS-Aβ42^2X^* and *UAS-Aβ42^BL33770^* have the signal peptide-encoding region of the fly *argos* gene, whereas *UAS-Aβ42^11C39^* and *UAS-Aβ42^H29.3^* have that of the rat *pre-proenkephalin* gene. *UAS-Aβ42^BL33770^* carries the poly A tail of *α-tubulin*, and the others contain that of *SV40*.

Based on well-developed genetic tools, such as the upstream activation sequence (*UAS*)*-GAL4* system, by which the expression of desired genes can be regulated, *Drosophila* has been used as an animal model to study AD (Lee et al. 2014, 2016; Bang et al. 2016). To date, different groups have generated several different transgenic lines that can be used for the ectopic expression of human *Aβ42*. In the present study, we selected four lines to investigate the relationship between transgene structure and their functions (Table 1 and Figure 1). *UAS-Aβ42^2X^* (Casas-Tinto et al. 2011) contains two serially concatenated copies of the transgene with an argos signal peptide and SV40 poly A tail (Figure 1). *UAS-Aβ42^BL33770^* (Singh & Mahoney 2011) contains one copy of the transgene with the argos signal peptide and the *Drosophila α-tubulin* 3′ untranslated region (UTR) (Figure 1). The *α-tubulin* 3′ UTR is thought to provide stability to transgenes linked with it (Ollmann et al. 2000; Liu et al. 2015); it is supposed to increase Aβ42 protein levels by prolonging the RNA half-life. *UAS-Aβ42^11C39^* (Iijima et al. 2008) and *UAS-Aβ42^H29.3^* (Finelli et al. 2004) both contain a copy of same transgene with a pre-proenkephalin signal peptide and an SV40 poly A tail (Figure 1).

**Table 1.** The list of studies in that used the *UAS-Aβ42* transgenic lines.

Line	Publication
2X	**Casas-Tinto et al. 2011. *Hum Mol Genet* 20(11):2144–2160**
	Ambegaokar & Jackson 2011. *Hum Mol Genet* 20(24):4947–4977
	Fernandez-Funez et al. 2015. *Hum Mol Genet* 24(21):6093–6105
BL33770	Liu et al. 2015. *Biol Pharm Bull* 38(12):1891–1901
	Lee et al. 2016. *Dis Model Mech* 9(3):295–306
	Chouhan et al. 2016. *Acta Neuropathol Commun* 4(1):62
	Liu et al. 2016. *Am J Chin Med* 44(7):1325–1347
11C39	**Iijima et al. 2008. *PLoS One* 3(2):e1703**
	Iijima-Ando et al. 2008. *J Biol Chem* 283(27):19066–19076
	Chiang et al. 2009. *FASEB J* 23(6):1969–1977
	Chiang et al. 2010. *Proc Natl Acad Sci USA* 107(15):7060–7065
	Iijima et al. 2010. *Hum Mol Genet* 19(15):2947–2957
	Lee et al. 2012. *Nat Commun* 3:1312
	Wang et al. 2012. *Proc Natl Acad Sci USA* 109(41):16743–16748
	Lang et al. 2012. *PLoS Genet* 8(4):e1002683
	Lang et al. 2013. *Neurobiol Aging* 34(11):2604–2612
	Lin et al. 2014. *Aging Cell* 13(3):507–518
	Ando et al. 2016. *PLoS Genet* 12(3):e1005917
H29.3	**Finelli et al. 2004. *Mol Cell Neurosci* 26(3):365–375**
	Cao et al. 2008. *Genetics* 178(3):1457–1471
	Ling et al. 2009. *PLoS One* 4(1):e4201
	Sanokawa-Akakura et al. 2010. *PLoS One* 5(1):e8626
	Ling & Salvaterra 2011. *Acta Neuropathol* 121(2):183–191
	Lee et al. 2011. *Mol Cells* 31(4):337–342
	Hong et al. 2012. *Biochem Biophys Res Commun* 419(1):49–53
	Lüchtenborg & Katanaev 2014. *Mol Brain* 7:81
	Liu et al. 2015. *Biol Pharm Bull* 38(12):1891–1901
	Lee et al. 2016. *Dis Model Mech* 9(3):295–306
	Gerstner et al. 2016. *J Neurosci Res* DOI:10.1002/jnr.23778
	Liu et al. 2016. *Am J Chin Med* 44(7):1325–1347

Although several *Drosophila Aβ42* transgenic lines were developed and used in a variety of studies, their phenotypic differences have not been studied in detail. Therefore, we compared the phenotypes of the four representative *UAS-Aβ42* lines under the same experimental conditions. They showed different *Aβ42* expression levels and phenotypic severity in eyes and neurons. Interestingly, the level of reactive oxygen species (ROS) generation did not correlate with survival rate in this comparative study.

## Materials and methods

### *Drosophila* strains

Glass multimer reporter (*GMR*)-*GAL4* (BL9146), embryonic lethal abnormal vision (*elav*)-*GAL4* (BL458), and *UAS-Aβ42^BL33770^* (BL33770) were acquired from the Bloomington *Drosophila* Stock Center. *UAS-Aβ42^2X^*, *UAS-Aβ42^H29.3^*, and *UAS-Aβ42^11C39^* were provided by Dr Pedro Fernandez-Funez (University of Florida, USA), Dr Mary Konsolaki (University of Rutgers, USA), and Dr Koichi M. Iijima (University of Thomas Jefferson, USA), respectively.

### Thioflavin S staining

Thioflavin S staining was performed as described previously by Iijima et al. (2004). Whole brains were dissected, permeabilized, and incubated overnight at 4°C in 50% ethanol containing 0.125% thioflavin S (Sigma-Aldrich). The samples were rinsed with 50% ethanol and phosphate buffered saline (PBS) containing 0.5% Triton X-100, and examined using confocal microscopy.

### Immunohistochemistry

Immunohistochemistry was performed as described previously by Jeong et al. (2015). Whole brains were dissected and blocked with 5% normal goat serum and 2% bovine serum albumin in PBS containing 0.5% Triton X-100. They were incubated for 48 h with anti-Aβ42 antibodies (1:200; Santa Cruz Biotechnology) at 4°C and washed four times with PBS containing 0.5% Triton X-100. Samples were then incubated overnight with Alexa-Fluor-488-labeled anti-mouse antibody (1:200; Invitrogen) at 4°C and washed four times with PBS containing 0.5% Triton X-100.

### Acridine Orange staining

Acridine orange (AO) staining was performed as described previously by Hong et al. (2012) and Park et al. (2013). The eye discs of stage L3 larvae were dissected rapidly in PBS and incubated for 5 min with 1.6 × 10^−6^ M AO (Sigma-Aldrich). After rinsing twice for 5 min in PBS, the samples were analyzed using a fluorescence microscope (Carl Zeiss).

### Dihydroethidium staining

For dihydroethidium (DHE) staining, the eye discs of stage L3 larvae were dissected in Schneider’s medium at room temperature, and incubated with Schneider’s medium containing the 3.0 × 10^−6^ M DHE dye (Invitrogen Molecular Probes) for 5 min in the dark. They were then washed with Schneider’s medium, and observed under a fluorescence microscope (Carl Zeiss).

### Analysis of *Drosophila* development

Fifty embryos of each genotype were collected in vials that contained standard cornmeal media and incubated at 25°C. Survival scores (the ratio of the number of adult male flies raised from collected embryos against half the total number of collected embryos) were obtained for each group. The experiment was repeated six times.

### Climbing assay

The climbing assay was performed as previously reported by Hwang et al. (2013) with some modifications. The experiment was conducted with 80 male flies. Ten male flies were collected in each climbing assay vial, and the flies were tapped down to the bottom of the vial. Then, the number of flies that climbed to the top of the vial within 15 s was counted. Ten trials were performed for each group. Climbing scores (the ratio of the number of flies that reached the top against the total number of flies) were obtained for each group, and the mean climbing scores for the 10 repeated tests were compared.

### Statistics

In all experiments, data were analyzed using one-way ANOVA followed by a Tukey–Kramer multiple comparison test. Statistical results were exhibited as means ± SEM. Decisive values were expressed by asterisks (*p < .05, **p < .01, and ***p < .001). Eye size was gauged using ImageJ software (National Institutes of Health).

## Results

### The levels of Aβ42 protein and its aggregates in the developing eyes and brains of *Drosophila* AD models

**Figure 2.**
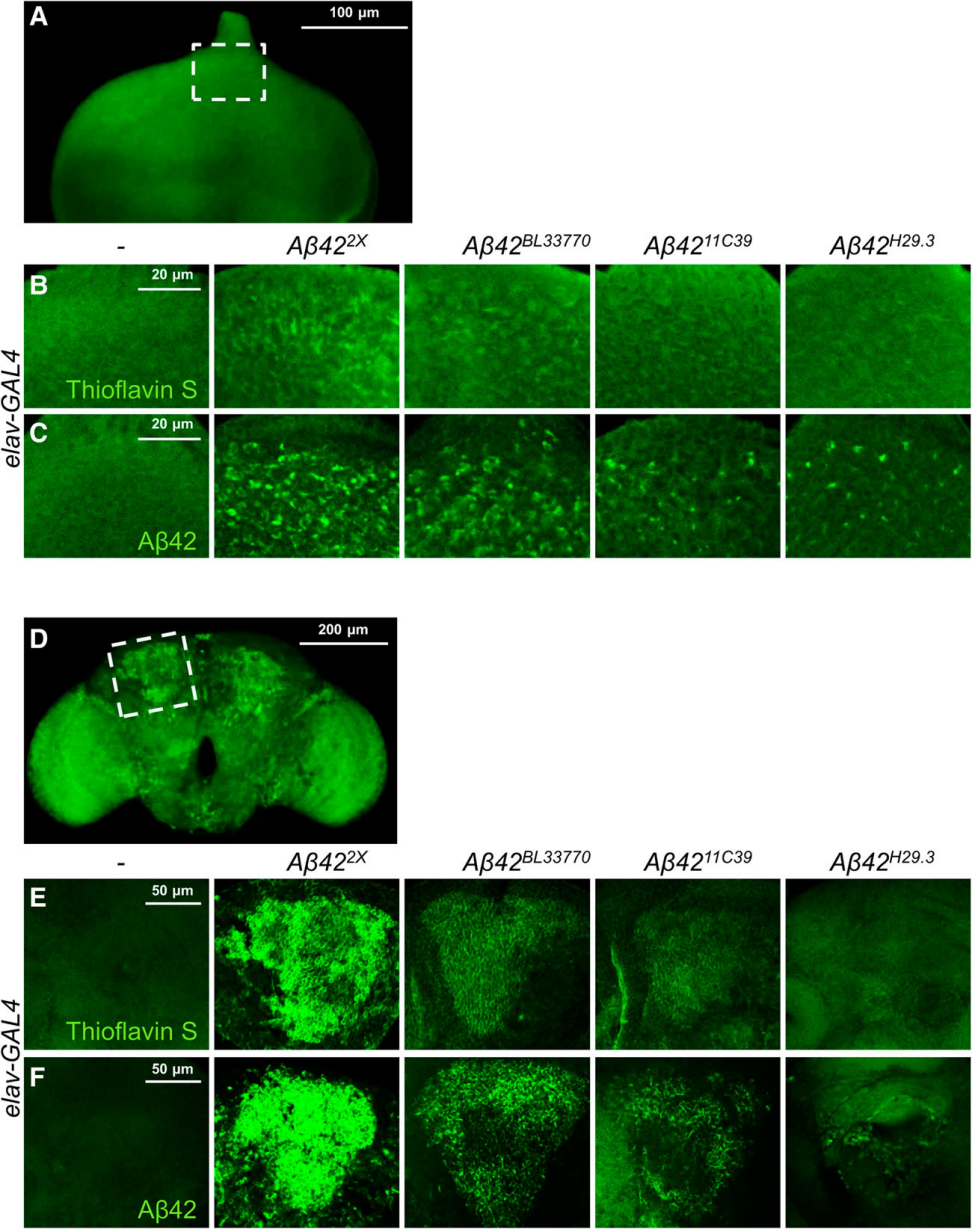
The Aβ42 levels and extent of aggregation in the eye imaginal discs (a–c) and the adult brains (d–f) of the flies expressing different *Aβ42* transgenes. Representative images of thioflavin S staining (a, b, d, e) and Aβ42-immunostaining (c, f) in eye imaginal discs and adult brains. (b) and (c) correspond to the dotted area in (a), while (e) and (f) correspond to the dotted area in (d). The human *Aβ42* transgenes were expressed in *Drosophila* eye imaginal discs at 29°C and neurons at 25°C, respectively. Magnification of the pictures: (a) ×200, (b, c, e, f) ×400, and (d) ×100.

To characterize the four different *Aβ42* transgenic lines, we measured the levels of Aβ42 aggregates and its protein abundance. As expected, the Aβ42 protein and its aggregation level in both the developing eyes and brain were the highest in the *UAS-Aβ42^2X^* line (Figure 2), which contains two copies of the *Aβ42* transgene (Figure 1). The second highest was the *UAS-Aβ42^BL33770^* line (Figure 2), which has an argos signal peptide and poly A tail of fly *α-tubulin* (Figure 1). The expression level of Aβ42 protein by the *UAS-Aβ42^11C39^* line was higher than that of *UAS-Aβ42^H29.3^* (Figure 2), despite having *Aβ42* transgenes with the same structure (Figure 1), which suggested that their difference might be caused by a position effect.

We also measured Aβ42 aggregate levels by thioflavin S staining, which is a commonly used method to detect amyloid fibrils, but not monomers (Yamamoto & Hirano 1986). The levels of Aβ42 aggregates were proportional to the protein levels (Figure 2), which indicated that the aggregation properties of the protein produced by the transgenes were similar.

### The levels of cell death induced by the four different *Aβ42* transgenes

**Figure 3.**
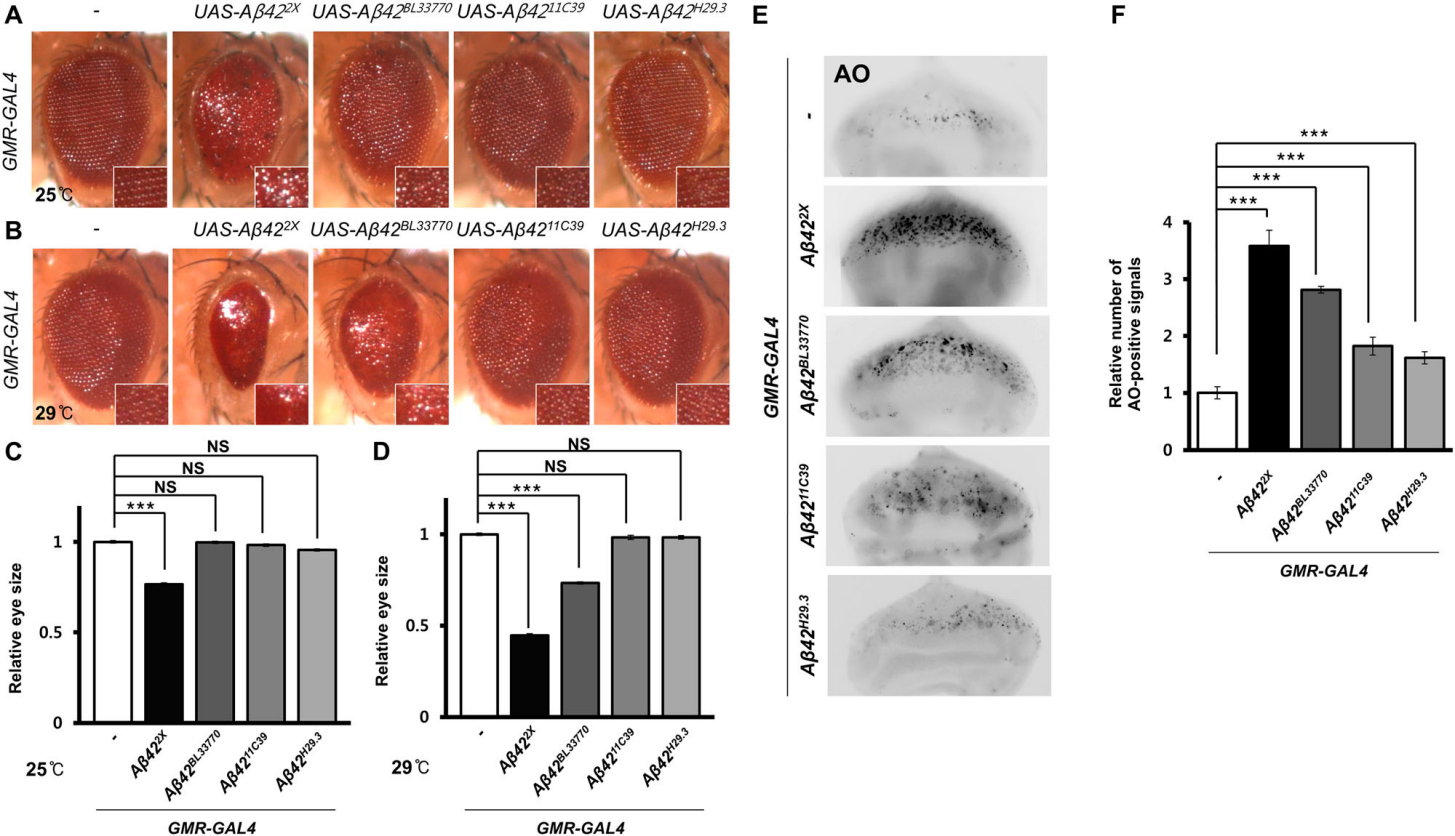
The morphology and cell death of *Drosophila* eyes expressing four *Aβ42* transgenes. (a, b) Pictures showing the eyes of flies expressing different *Aβ42* transgenes at different temperatures. Magnification of the pictures, ×50. (c, d) Graphs showing the relative sizes of the eyes of each experimental group (Tukey–Kramer test, *n* ≥ 19, ***p < .001, NS, not significant). (e) Fluorescent microscopic images of AO-stained eye imaginal discs expressing human *Aβ42* using four different transgenic lines at 29°C. Magnification of the pictures, ×200. (f) A graph showing the relative number of AO-positive signals in the eye imaginal disc of each experimental group (Tukey–Kramer test, *n* ≥ 17, ***p < .001).

Next, we examined the cell death induced by the transgenes in developing eyes, which have been used frequently to measure cell death (Lee et al. 2014). Ectopic *Aβ42* expression resulted in severely reduced and rough eyes in the *UAS-Aβ42^2X^* lines when reared at both 25°C and 29°C (Figure 3(a)–(d)). However, the eye phenotype of flies expressing the *UAS-Aβ42^BL33770^* transgene depended on the rearing temperature. The reduced and rough eye phenotype appeared only at 29°C, while the rough eye phenotype without size reduction appeared at 25°C (Figure 3(a)–(d)). The flies expressing *UAS-Aβ42^11C39^* and *UAS-Aβ42^H29.3^* showed very mild rough eye phenotype at 29°C (Figure 3(b)). The number of dead cells in the developing eyes was consistently the highest in *UAS-Aβ42^2X^*, followed by *UAS-Aβ42^BL33770^*, *UAS-Aβ42^11C39^*, and *UAS-Aβ42^H29.3^* (Figure 3(e) and 3(f)).

### The levels of ROS in the flies expressing the four different *Aβ42* transgenes

**Figure 4.**
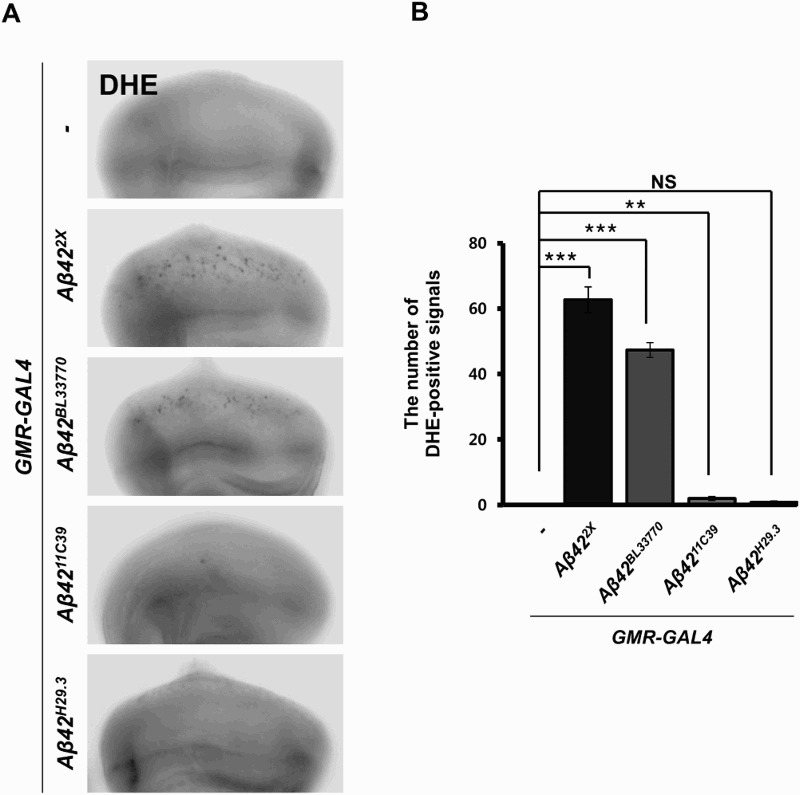
The ROS levels in the flies expressing four different *Aβ42* transgenes. (a) Fluorescent microscopic images of DHE-stained eye imaginal discs expressing human *Aβ42* using four different transgenic lines at 29°C. Magnification of the pictures, ×200. (b) A graph showing ROS levels, which were detected by DHE staining (Tukey–Kramer test, *n* ≥ 18, **p < .01, ***p < .001; NS, not significant).

ROS generation is an important pathological characteristic of AD, and ROS is closely associated with neuronal cell death (Markesbery 1997); therefore, we also measured the ROS levels using DHE staining in the eye imaginal discs expressing the *Aβ42* transgenes. A prominent amount of ROS was detected in the eye imaginal discs expressing *UAS-Aβ42^2X^* and *UAS-Aβ42^BL33770^*, while little was observed in the discs expressing *UAS-Aβ42^11C39^* and *UAS-Aβ42^H29.3^* (Figure 4).

### The phenotypes of the flies expressing the *Aβ42* transgenes in neurons

**Figure 5.**
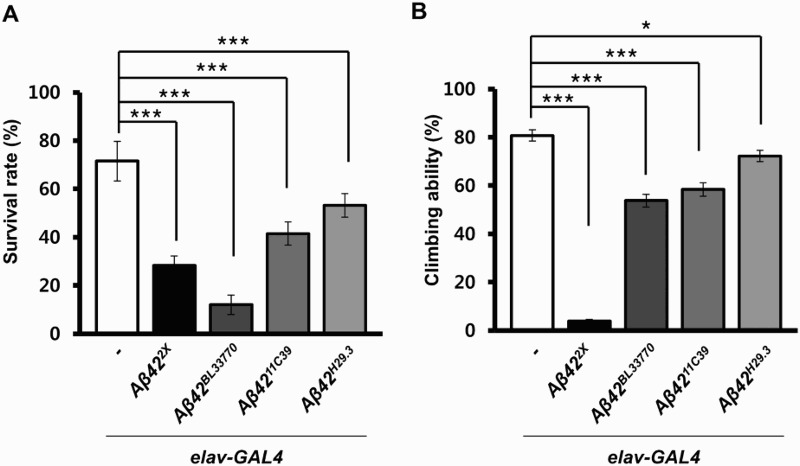
Survival rates and climbing ability of neuronal *Aβ42*-expressing flies with four different *Aβ42* transgenes. (a) A graph showing the survival rates of *Drosophila* expressing human *Aβ42* in their brains using four different transgenic lines at 25°C (Tukey–Kramer test, *n* ≥ 180, ***p < .001). (b) A graph showing the climbing ability of *Aβ42*-expressing flies at 25°C (Tukey–Kramer test, *n* ≥ 80, *p < .05, ***p < .001).

We also examined the effects of transgene expression in neurons during development by calculating the survival rates, which were the ratio of emerged adults from eggs. Interestingly, the trend of decreased survival in each *Aβ42-*expressing line was different from the levels of *Aβ42* expression or the eye phenotype (Figure 5(a)). The survival rate of the *UAS-Aβ42^BL33770^* flies was the lowest, while the *UAS-Aβ42^11C39^* and *UAS-Aβ42^H29.3^* flies also showed significantly reduced survival (Figure 5(a)).

To compare the effects of *Aβ42* expression on adult neurological function, the locomotor activities of the flies expressing the transgenes were measured. Surprisingly, the trend in the locomotor dysfunction levels in the *UAS-Aβ42^2X^* and *UAS-Aβ42^BL33770^* lines was quite different from that of their survival rates (Figure 5(b)). Although the survival rate of *UAS-Aβ42^BL33770^* flies was extremely low (12%), upon emerging, they only showed a moderate locomotor defect (Figure 5(b)), which suggested that the surviving flies may be relatively healthy.

## Discussion

In this study, we compared the expression levels of *Aβ42* and the phenotypes of flies expressing four frequently used *UAS-Aβ42* transgenes. The relative expression levels of *Aβ42* in the transgenic lines are similar in both the developing eyes and brain. Both the Aβ42 proteins and its aggregation levels were consistently the highest in the developing eyes and brain of *UAS-Aβ42^2X^* line, followed by *UAS-Aβ42^BL33770^*, *UAS-Aβ42^11C39^*, and *UAS-Aβ42^H29.3^*. However, the effects of *Aβ42* expression on the phenotypes in these lines were different in these tissues. The eyes of flies expressing *UAS-Aβ42^2X^* or *UAS-Aβ42^BL33770^* showed severe defects, while *UAS-Aβ42^11C39^* or *UAS-Aβ42^H29.3^* flies had very mild rough eye phenotypes, which correlated with Aβ42 protein levels. In contrast, the severity of neuronal phenotypes in each transgenic line did not correlate with Aβ42 protein levels. When the transgenes were expressed pan-neuronally using the *elav-GAL4* driver, the survival rate was reduced significantly in both *UAS-Aβ42^11C39^* and *UAS-Aβ42^H29.3^*, unlike their eye phenotypes. This discrepancy in the effects of Aβ42 in the different tissues might be caused by the difference in susceptibility between neurons and non-neuronal cells. In support of this notion, a previous study showed that Aβ oligomer administration induced cell death in primary cultures of rat cortical neurons, but not in astrocytes (Ebenezer et al. 2010). The hypersensitivity of neuronal cells to Aβ oligomers might reflect the high level of Aβ oligomer receptors, such as the receptor for advanced glycation end products (Du Yan et al. 1996) and prions (Laurén et al. 2009), or erroneous cell cycle activation by the Aβ protein in neurons (Caricasole et al. 2003). Although the detailed mechanism is not clear, our data suggest that the Aβ hypersensitivity of neuronal cells is conserved in *Drosophila*.

We also found that the survival rate of the flies expressing *UAS-Aβ42^BL33770^* in neurons was the lowest, while *Aβ42* expression levels of these flies are much lower than that of flies expressing *UAS-Aβ42^2X^* transgene. This result suggests that the neurotoxicity of Aβ42 is not simply determined by Aβ42 levels. This phenomenon is also well known in human brain. That is, the degree of cognitive impairment in AD patients does not correlate well with the brain Aβ deposits number (Hardy & Selkoe 2002). However, the soluble Aβ concentrations were inversely correlated with synapse loss in AD patients and distinguished AD patients from high pathology control patients (Lue et al. 1999), which suggests that soluble Aβ42 oligomers, but not insoluble Aβ42 deposits, are responsible for AD pathology such as synapse loss. Therefore, the unexpected highly decreased survival rate of *Aβ42^BL33770^*-expressing flies would be the result from the high level of soluble Aβ42 oligomer generation in this line.

The difference between the constructs of the transgenes in different *UAS-Aβ42* lines might also be associated with their phenotypic variation. The different secretory abilities of the Aβ42 peptide expressed from each transgenic line might explain the unexpected strong reduction of survival during the development of flies expressing *UAS-Aβ42^11C39^* or *UAS-Aβ42^H29.3^* in neurons. As these lines contain a mammalian signal peptide, Aβ42 proteins might be secreted less efficiently in these lines compared to *UAS-Aβ42^2X^* and *UAS-Aβ42^BL33770^* lines, which contain a *Drosophila* signal peptide. In that case, flies with *UAS-Aβ42^11C39^* and *UAS-Aβ42^H29.3^* might secrete little Aβ42 out of the cells, resulting in intracellular Aβ42 accumulation that would damage mitochondria. In contrast, the *UAS-Aβ42^2X^* and *UAS-Aβ42^BL33770^* lines secreted most of the Aβ42 proteins outside the cells, while relatively little accumulates in the cytoplasm. Further studies on the Aβ42 secretion for each transgenic line are needed to reveal the detailed mechanism of Aβ42 cytotoxicity.

In addition, the effect of different genetic backgrounds should be considered. Although we used the same *GAL4* lines to express the four different *Aβ42* transgenes ectopically, the transgenic lines have different genetic backgrounds, which could affect the phenotypes produced by the transgenes. Therefore, to exclude this possibility completely, further studies should be conducted with the new transgenes with clear genetic backgrounds, which can be achieved by backcrossing to the same control line, such as *w^1118^*.

In conclusion, our data demonstrate that different *Drosophila* AD models show different phenotypic severity in different tissues, and suggest that different *Aβ42* transgenes might have different modes of cytotoxicity. Therefore, AD models should be designed for the specific aims of each study.

## References

[CIT0001] Ambegaokar SS, Jackson GR. 2011 Functional genomic screen and network analysis reveal novel modifiers of tauopathy dissociated from tau phosphorylation. Hum Mol Genet. 20:4947–4977. doi: 10.1093/hmg/ddr432 21949350 PMC3221533

[CIT0002] Ando K, Maruko-Otake A, Ohtake Y, Hayashishita M, Sekiya M, Iijima KM. 2016 Stabilization of microtubule-unbound Tau via Tau phosphorylation at Ser262/356 by Par-1/MARK contributes to augmentation of AD-related phosphorylation and Aβ42-induced Tau toxicity. PLoS Genet. 12:e1005917. doi: 10.1371/journal.pgen.1005917 27023670 PMC4811436

[CIT0003] Bang SM, Lee S, Jeong H, Hong YK, Lee JH, Hwang S, Suh YS, Lee K, Cho KS. 2016 Effects of sarah/nebula knockdown on Aβ42-induced phenotypes during *Drosophila* development. Genes Genomics. 38:479–487. doi: 10.1007/s13258-016-0407-5

[CIT0004] Berridge MJ. 2010 Calcium hypothesis of Alzheimer’s disease. Pflugers Arch. 459:441–449. doi: 10.1007/s00424-009-0736-1 19795132

[CIT0005] Cao W, Song HJ, Gangi T, Kelkar A, Antani I, Garza D, Konsolaki M. 2008 Identification of novel genes that modify phenotypes induced by Alzheimer’s β-amyloid overexpression in *Drosophila*. Genetics. 178:1457–1471. doi: 10.1534/genetics.107.078394 18245849 PMC2278065

[CIT0006] Caricasole A, Copani A, Caruso A, Caraci F, Iacovelli L, Sortino MA, Terstappen GC, Nicoletti F. 2003 The Wnt pathway, cell-cycle activation and β-amyloid: novel therapeutic strategies in Alzheimer’s disease? Trends Pharmacol Sci. 24:233–238. doi: 10.1016/S0165-6147(03)00100-7 12767722

[CIT0007] Casas-Tinto S, Zhang Y, Sanchez-Garcia J, Gomez-Velazquez M, Rincon-Limas DE, Fernandez-Funez P. 2011 The ER stress factor XBP1s prevents amyloid-β neurotoxicity. Hum Mol Genet. 20:2144–2160. doi: 10.1093/hmg/ddr100 21389082 PMC3090193

[CIT0008] Chiang HC, Iijima K, Hakker I, Zhong Y. 2009 Distinctive roles of different β-amyloid 42 aggregates in modulation of synaptic functions. FASEB J. 23:1969–1977. doi: 10.1096/fj.08-121152 19255256 PMC2698658

[CIT0009] Chiang HC, Wang L, Xie Z, Yau A, Zhong Y. 2010 PI3 kinase signaling is involved in Aβ-induced memory loss in *Drosophila*. Proc Natl Acad Sci U S A. 107:7060–7065. doi: 10.1073/pnas.0909314107 20351282 PMC2872421

[CIT0010] Chouhan AK, Guo C, Hsieh YC, Ye H, Senturk M, Zuo Z, Li Y, Chatterjee S, Botas J, Jackson GR, et al. 2016 Uncoupling neuronal death and dysfunction in *Drosophila* models of neurodegenerative disease. Acta Neuropathol Commun. 4:62. doi: 10.1186/s40478-016-0333-4 27338814 PMC4918017

[CIT0011] Du Yan S, Chen X, Fu J, Chen M, Zhu H, Roher A, Slattery T, Zhao L, Nagashima M, Morser J, et al. 1996 RAGE and amyloid-β peptide neurotoxicity in Alzheimer’s disease. Nature. 382:685–691. doi: 10.1038/382685a0 8751438

[CIT0012] Ebenezer PJ, Weidner AM, LeVine H, Markesbery WR, Murphy MP, Zhang L, Dasuri K, Fernandez-Kim SO, Bruce-Keller AJ, Gavilan E, et al. 2010 Neuron specific toxicity of oligomeric amyloid-beta: role for JUN-kinase and oxidative stress. J Alzheimers Dis. 22:839–848. doi: 10.3233/JAD-2010-101161 20858948 PMC3412400

[CIT0013] Fernandez-Funez P, Zhang Y, Sanchez-Garcia J, de Mena L, Khare S, Golde TE, Levites Y, Rincon-Limas DE. 2015 Anti-Aβ single-chain variable fragment antibodies exert synergistic neuroprotective activities in *Drosophila* models of Alzheimer’s disease. Hum Mol Genet. 24:6093–6105. doi: 10.1093/hmg/ddv321 26253732 PMC4599669

[CIT0014] Finelli A, Kelkar A, Song H-J, Yang H, Konsolaki M. 2004 A model for studying Alzheimer’s Aβ42-induced toxicity in *Drosophila melanogaster*. Mol Cell Neurosci. 26:365–375. doi: 10.1016/j.mcn.2004.03.001 15234342

[CIT0015] Francis PT, Palmer AM, Snape M, Wilcock GK. 1999 The cholinergic hypothesis of Alzheimer’s disease: a review of progress. J Neurol Neurosurg Psychiatr. 66:137–147. doi: 10.1136/jnnp.66.2.137 PMC1736202 10071091

[CIT0016] Gerstner JR, Lenz O, Vanderheyden WM, Chan MT, Pfeiffenberger C, Pack AI. 2016 Amyloid-β induces sleep fragmentation that is rescued by fatty acid binding proteins in *Drosophila*. J Neurosci Res. 10.1002/jnr.23778. 10.1002/jnr.23778 PMC5167666 27320125

[CIT0017] Hardy J, Selkoe DJ. 2002 The amyloid hypothesis of Alzheimer’s disease: progress and problems on the road to therapeutics. Science. 297:353–356. doi: 10.1126/science.1072994 12130773

[CIT0018] Hardy JA, Higgins GA. 1992 Alzheimer's disease: the amyloid cascade hypothesis. Science. 256:184–185. doi: 10.1126/science.1566067 1566067

[CIT0019] Hong YK, Lee S, Park SH, Lee JH, Han SY, Kim ST, Kim Y-K, Jeon S, Koo B-S, Cho KS. 2012 Inhibition of JNK/dFOXO pathway and caspases rescues neurological impairments in *Drosophila* Alzheimer’s disease model. Biochem Biophys Res Commun. 419:49–53. doi: 10.1016/j.bbrc.2012.01.122 22326868

[CIT0020] Hwang S, Song S, Hong YK, Choi G, Suh YS, Han SY, Lee M, Park SH, Lee JH, Lee S, et al. 2013 *Drosophila* DJ-1 decreases neural sensitivity to stress by negatively regulating Daxx-like protein through dFOXO. PLoS Genet. 9:e1003412. doi: 10.1371/journal.pgen.1003412 23593018 PMC3616925

[CIT0021] Iijima-Ando K, Hearn SA, Granger L, Shenton C, Gatt A, Chiang H-C, Hakker I, Zhong Y, Iijima K. 2008 Overexpression of neprilysin reduces Alzheimer amyloid-β42 (Aβ42)-induced neuron loss and intraneuronal Aβ42 deposits but causes a reduction in cAMP-responsive element-binding protein-mediated transcription, age-dependent axon pathology, and premature death in *Drosophila*. J Biol Chem. 283:19066–19076. doi: 10.1074/jbc.M710509200 18463098 PMC2441542

[CIT0022] Iijima K, Chiang H-C, Hearn SA, Hakker I, Gatt A, Shenton C, Granger L, Leung A, Iijima-Ando K, Zhong Y. 2008 *Aβ42* mutants with different aggregation profiles induce distinct pathologies in *Drosophila*. PloS One. 3:e1703. doi: 10.1371/journal.pone.0001703 18301778 PMC2250771

[CIT0023] Iijima K, Gatt A, Iijima-Ando K. 2010 Tau Ser262 phosphorylation is critical for Aβ42-induced tau toxicity in a transgenic *Drosophila* model of Alzheimer’s disease. Hum Mol Genet. 19:2947–2957. doi: 10.1093/hmg/ddq200 20466736 PMC2901137

[CIT0024] Iijima K, Liu HP, Chiang AS, Hearn SA, Konsolaki M, Zhong Y. 2004 Dissecting the pathological effects of human Abeta40 and Abeta42 in *Drosophila*: a potential model for Alzheimer’s disease. Proc Natl Acad Sci U S A. 101:6623–6628. doi: 10.1073/pnas.0400895101 15069204 PMC404095

[CIT0025] Jeong H, Han SY, Lee M, Lee S, Shin M, Jeon Y, Lee K, Cho KS. 2015 Roles of Tsp66E and Tsp74F in border cell migration and the maintenance of border cell adhesion in *Drosophila*. Genes Genomics. 37:559–565. doi: 10.1007/s13258-015-0285-2

[CIT0026] Lang M, Fan Q, Wang L, Zheng Y, Xiao G, Wang X, Wang W, Zhong Y, B Z. 2013 Inhibition of human high-affinity copper importer Ctr1 orthologous in the nervous system of *Drosophila* ameliorates Abeta42-induced Alzheimer’s disease-like symptoms. Neurobiol Aging. 34:2604–2612. doi: 10.1016/j.neurobiolaging.2013.05.029 23827522 PMC3770863

[CIT0027] Lang M, Wang L, Fan Q, Xiao G, Wang X, Zhong Y, Zhou B. 2012 Genetic inhibition of solute-linked carrier 39 family transporter 1 ameliorates aβ pathology in a *Drosophila* model of Alzheimer’s disease. PLoS Genet. 8:e1002683. doi: 10.1371/journal.pgen.1002683 22570624 PMC3343105

[CIT0028] Laurén J, Gimbel DA, Nygaard HB, Gilbert JW, Strittmatter SM. 2009 Cellular prion protein mediates impairment of synaptic plasticity by amyloid-beta oligomers. Nature. 457:1128–1132. doi: 10.1038/nature07761 19242475 PMC2748841

[CIT0029] Lee MJ, Park SH, Han JH, Hong YK, Hwang S, Lee S, Kim D, Han SY, Kim ES, Cho KS. 2011 The effects of hempseed meal intake and linoleic acid on *Drosophila* models of neurodegenerative diseases and hypercholesterolemia. Mol Cells. 31:337–342. doi: 10.1007/s10059-011-0042-6 21331775 PMC3933972

[CIT0030] Lee S, Bang SM, Hong YK, Lee JH, Jeong H, Park SH, Liu QF, Lee IS, Cho KS. 2016 The calcineurin inhibitor sarah (nebula) exacerbates Abeta42 phenotypes in a *Drosophila* model of Alzheimer’s disease. Dis Model Mech. 9:295–306. doi: 10.1242/dmm.018069 26659252 PMC4826976

[CIT0031] Lee S, Bang SM, Lee JW, Cho KS. 2014 Evaluation of traditional medicines for neurodegenerative diseases using *Drosophila* models. Evid Based Complement Alternat Med. eCAM.2014:967462. 10.1155/2014/967462 PMC3984789 24790636

[CIT0032] Lee S, Wang JW, Yu W, Lu B. 2012 Phospho-dependent ubiquitination and degradation of PAR-1 regulates synaptic morphology and tau-mediated Aβ toxicity in *Drosophila*. Nat Commun. 3:1312. doi: 10.1038/ncomms2278 23271647 PMC4307937

[CIT0033] Lin R, Angelin A, Da Settimo F, Martini C, Taliani S, Zhu S, Wallace DC. 2014 Genetic analysis of dTSPO, an outer mitochondrial membrane protein, reveals its functions in apoptosis, longevity, and Aβ42-induced neurodegeneration. Aging Cell. 13:507–518. doi: 10.1111/acel.12200 24977274 PMC4076708

[CIT0034] Ling D, Salvaterra PM. 2011 Brain aging and Aβ1–42 neurotoxicity converge via deterioration in autophagy–lysosomal system: a conditional *Drosophila* model linking Alzheimer’s neurodegeneration with aging. Acta Neuropathol. 121:183–191. doi: 10.1007/s00401-010-0772-0 21076961

[CIT0035] Ling D, Song H-J, Garza D, Neufeld TP, Salvaterra PM. 2009 Abeta42-induced neurodegeneration via an age-dependent autophagic-lysosomal injury in *Drosophila*. PLoS One. 4:e4201. doi: 10.1371/journal.pone.0004201 19145255 PMC2626277

[CIT0036] Liu QF, Jeong H, Lee JH, Hong YK, Oh Y, Kim YM, Suh YS, Bang S, Yun HS, Lee K, et al. 2016 *Coriandrum sativum* suppresses Aβ42-induced ROS increases, glial cell proliferation, and ERK activation. Am J Chin Med. 44:1325–1347. doi: 10.1142/S0192415X16500749 27776428

[CIT0037] Liu QF, Lee JH, Kim Y-M, Lee S, Hong YK, Hwang S, Oh Y, Lee K, Yun HS, Lee IS, et al. 2015 In vivo screening of traditional medicinal plants for neuroprotective activity against Aβ42 cytotoxicity by using *Drosophila* models of Alzheimer’s disease. Biol Pharm Bull. 38:1891–1901. doi: 10.1248/bpb.b15-00459 26458335

[CIT0038] Lüchtenborg AM, Katanaev VL. 2014 Lack of evidence of the interaction of the Aβ peptide with the Wnt signaling cascade in *Drosophila* models of Alzheimer’s disease. Mol Brain. 7:81. 25387847 10.1186/s13041-014-0081-y PMC4232725

[CIT0039] Lue LF, Kuo YM, Roher AE, Brachova L, Shen Y, Sue L, Beach T, Kurth JH, Rydel RE, Rogers J. 1999 Soluble amyloid β peptide concentration as a predictor of synaptic change in Alzheimer’s disease. Am J Pathol. 155:853–862. doi: 10.1016/S0002-9440(10)65184-X 10487842 PMC1866907

[CIT0040] Maccioni RB, Farias G, Morales I, Navarrete L. 2010 The revitalized tau hypothesis on Alzheimer’s disease. Arch Med Res. 41:226–231. doi: 10.1016/j.arcmed.2010.03.007 20682182

[CIT0041] Markesbery WR. 1997 Oxidative stress hypothesis in Alzheimer’s disease. Free Radic Biol Med. 23:134–147. doi: 10.1016/S0891-5849(96)00629-6 9165306

[CIT0042] Mattson MP. 2004 Pathways towards and away from Alzheimer’s disease. Nature. 430:631–639. doi: 10.1038/nature02621 15295589 PMC3091392

[CIT0043] Ollmann M, Young LM, Di Como CJ, Karim F, Belvin M, Robertson S, Whittaker K, Demsky M, Fisher WW, Buchman A, et al. 2000 *Drosophila* p53 is a structural and functional homolog of the tumor suppressor p53. Cell. 101:91–101. doi: 10.1016/S0092-8674(00)80626-1 10778859

[CIT0044] Park SH, Lee S, Hong YK, Hwang S, Lee JH, Bang SM, Kim YK, Koo BS, Lee IS, Cho KS. 2013 Suppressive effects of SuHeXiang Wan on amyloid-β42-induced extracellular signal-regulated kinase hyperactivation and glial cell proliferation in a transgenic *Drosophila* model of Alzheimer’s disease. Biol Pharm Bull. 36:390–398. doi: 10.1248/bpb.b12-00792 23238278

[CIT0045] Sanokawa-Akakura R, Cao W, Allan K, Patel K, Ganesh A, Heiman G, Burke R, Kemp FW, Bogden JD, Camakaris J, et al. 2010 Control of Alzheimer’s amyloid beta toxicity by the high molecular weight immunophilin FKBP52 and copper homeostasis in *Drosophila*. PLoS One. 5:e8626. doi: 10.1371/journal.pone.0008626 20084280 PMC2801609

[CIT0046] Singh C, Mahoney M. 2011 *UAS-APP and APP-based constructs and insertions from Vitruvean. Flybase*. Personal communication to Flybase:FBrf0213105.

[CIT0047] Wang L, Chiang HC, Wu W, Liang B, Xie Z, Yao X, Ma W, Du S, Zhong Y. 2012 Epidermal growth factor receptor is a preferred target for treating amyloid-β-induced memory loss. Proc Natl Acad Sci U S A. 109:16743–16748. doi: 10.1073/pnas.1208011109 23019586 PMC3478595

[CIT0048] Yamamoto T, Hirano A. 1986 A comparative study of modified Bielschowsky, Bodian and thioflavin S stains on Alzheimer’s neurofibrillary tangles. Neuropathol Appl Neurobiol. 12:3–9. doi: 10.1111/j.1365-2990.1986.tb00677.x 2422580

